# Design and Preliminary Ground Experiment for Deployable Sunshade Structures of a Modular Space Telescope

**DOI:** 10.3390/s24072280

**Published:** 2024-04-03

**Authors:** Ye Kuang, Shuaihui Wang, Yan Gao, Boqian Xu, Shuyan Xu

**Affiliations:** 1 Changchun Institute of Optics, Fine Mechanics and Physics, Chinese Academy of Sciences, Changchun 130033, China; wangshuaihui@ciomp.ac.cn (S.W.); gaoyan@ciomp.ac.cn (Y.G.); xuboqian@ciomp.ac.cn (B.X.); xusy@ciomp.ac.cn (S.X.); 2 Chinese Academy of Sciences Key Laboratory of On-Orbit Manufacturing and Integration for Space Optics System, Changchun 130033, China; 3 University of Chinese Academy of Sciences, Beijing 100049, China

**Keywords:** deployable sunshade, modular space telescope, on-orbit assembling space telescope

## Abstract

On-orbit assembling space telescope (OAST) is one of the most feasible methods to implement a large-scale space telescope. Unlike a monolithic space telescope (such as Hubble Space Telescope, HST) or a deployable space telescope (such as James Webb Space Telescope, JWST), OAST can be assembled in the spatial environment. To ensure proper telescope performance, OAST must be equipped with a large deployable sunshade. In order to verify the technology of the OAST, the authors propose a modular space telescope on the China Space Station (CSS) and design a deployable sunshade. The deployable mechanism of the sunshade is made up of a radial deployable mechanism and an axial deployable mechanism. The paper describes the overall design approach, the key component technologies, and the design and preliminary testing of a part of the deployable sunshade assembly.

## 1. Introduction

Space telescopes play an important role in astronomical research. They are the key platforms for the astronomy study. According to the optical principle, increasing the optical aperture can significantly increase the observation angle resolution. To meet the growing demands of multiple applications, larger-aperture space telescopes need to be built in the future. A simple way to increase the optical aperture is to make the primary mirror bigger. One way is to design an on-orbit assembling space telescope (OAST).

The Next Generation Space Telescope started in 1996, and in 2002, it was renamed the James Webb Space Telescope (JWST) [1]. It could compress its space by folding the primary mirror, second mirror, sunshade, and so on into the Ariane V ECA fairing and deployed in the space. The primary mirror diameter of the JWST is 6.5 m. The next space telescope, such as LUVOIR [2], has the same way to fold the telescope into the SLS. But this approach uses a single launching vehicle, which has a certain carrying capacity.

The OAST was first presented by NASA around the early 2000s. It has a modular design and can be modularly manufactured on the ground and modularly launched into space. The OAST can be launched by using several carrier rockets, unlike the deployable space telescope that only uses one rocket.

**Figure 1:**
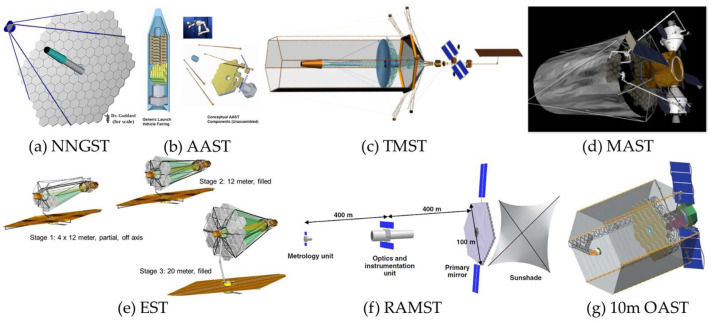
Several OAST projects.

Several assembling space telescope projects have been put forward, as shown in Figure 1. In 1999, Boeing had a space telescope project named the Next, Next Generation Space Telescope (NNGST) [3]. Then, the autonomously assembled space telescope (AAST) was brought out in 2003 [4]. NASA and GFSC introduced the Thirty Meter Space Telescope (TMST) in 2006 [5] and the Modular Assembled Space Telescope (MAST) in 2013 [6]. The evolvable space telescope (EST) project was planned, with three assembly stages [7,8]. In 2015, JPL brought out the robotically assembled, modular space telescope (RAMST) [9]. CIOMP is coming up with a 10-m-diameter OAST [10,11].

**Figure 2:**
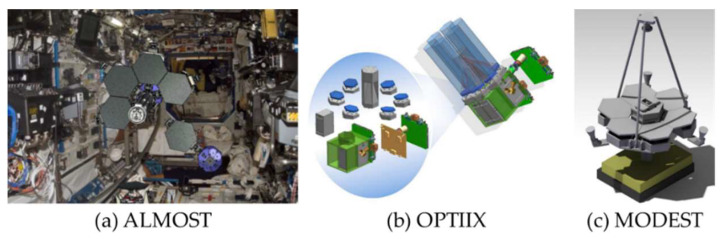
The small OAST projects.

To verify the technology of the OAST, several small OASTs have been brought out, such as the assembly of a large modular optical space telescope (ALMOST) in 2008 [12], Optical Testbed and Integration on ISS eXperiment (OPTIIX) in 2012 [13], and modular orbital demonstration of an evolvable space telescope (MODEST) in 2015 [8,14], as shown in Figure 2. The small OASTs described above are both planned for the International Space Station.

In addition to the telescopes mentioned above, antennas also can be deployed in space. Antennas with a diameter of 100 m and larger were designed in the 1970s [15,16,17], as well as other antennas, like Sunflower [18], MEA [18], SSDA [19], and EGS [20]. But they have a paraboloid surface, not suitable for the OAST.

At present, the China Space Station (CSS) has completed several launches. Tianhe core module, Wentian lab module, and Mengtian lab module have completed joint work. In the future, the China Space Station Telescope (CSST) will be launched into space. Soon, the OAST technology can be verified in space.

The deployable sunshade is a key technology for the OAST. The telescopes need to have a large deployable sunshade structure design, such as on the JWST [21,22,23,24], LUVOIR [2], and IXO [25]. In this paper, we demonstrate the design and analysis of the sunshade of the OAST on the CSS. We hope some of the analysis results will be helpful to the design of the OAST and other space telescopes.

## 2. The Modular Space Telescope on CSS

### 2.1. The Top Design of the Modular Space Telescope on CSS

**Figure 3:**
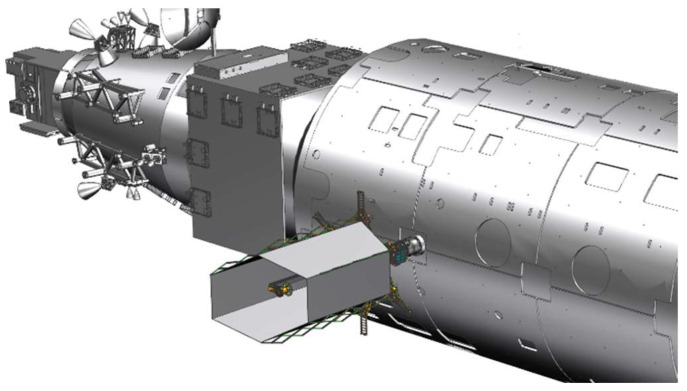
The modular space telescope’s working state on the CSS.

Like the OPTIIX and MODEST projects, the modular space telescope modules can be transported to the CSS by cargo ships. The modular space telescope can be assembled by robotic arms or astronauts. The modular space telescope consists of several modular components, such as an optical module, submirror module, second mirror module, sunshade, etc. The modular space telescope’s working state is shown in Figure 3.

Based on existing engineering experience, such as JWST, to reduce the impact of external vibration, the sunshade is mounted on the bus. There are also vibration isolation devices between the optical system and the bus. To keep the quality of the imaging, the space telescope modules need vibration isolation devices. However, the sunshade cannot be put on the CSS, so the vibration isolation devices can only be put at the bottom of the optical system, which reduces the external vibration effects from the sunshade and CSS as much as possible.

### 2.2. The Ground Experiment of the Robot Assembly Process

In the early work, the assembly test of the prototype of the ground telescope was carried out. The whole test system is composed of a structural prototype, a submirror module, a second mirror module, and a robotic arm.

**Figure 4:**
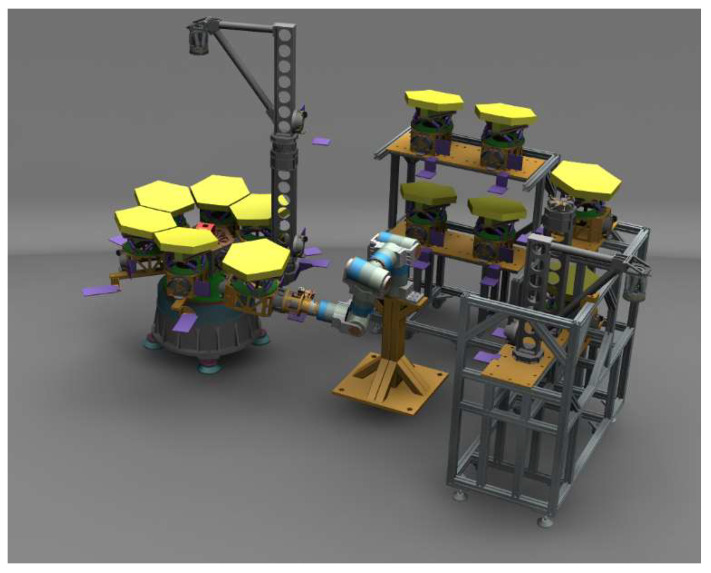
The simulation of the experimental process for submirror assembly.

**Figure 5:**
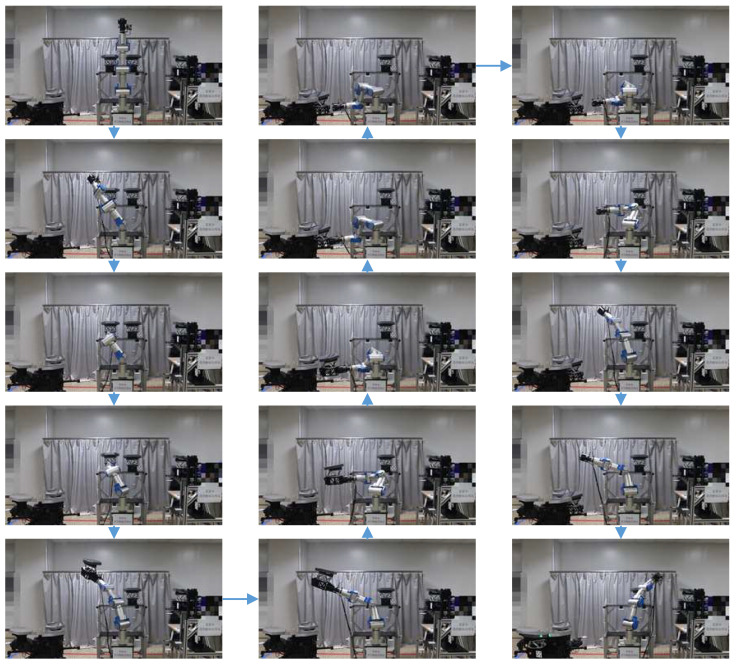
The experimental process for submirror assembly.

Through 3D software (UG 10), we simulate the assembly process and determine the relative position relationship of the robotic arm and telescope prototype. The assembly simulation is shown in Figure 4. Then, the actual assembly test is carried out, and the assembly is shown in Figure 5.

In the process of the submirror assembly, the robotic arm first grabs the submirror. According to the spatial trajectory planning, the robotic arm moves the submirror to the installation location. Using the hand-eye camera, which is at the end of the robotic arm, the robotic arm completes the approach to the telescope prototype. When the submirror interface moves to the capture range, the docking interface is actively locked and the robotic arm moves through the force sensor. After the prototype lock is completed, the robotic arm unlocks the submirror and moves away from the telescope prototype. The telescope prototype will then rotate to prepare for the next assembly.

## 3. The Design of the Deployable Sunshade Assembly Structures

### 3.1. Requirements

After the optical elements are manufactured, the space telescope modules are transported to the CSS by cargo ships. Astronauts will check modules and complete parts of the assembly work in the CSS. The main optical machine module can be moved to the outer load of the CSS cabin by the robotic arm.

To verify the assembly process of the module, the module installation is completed by using the robotic arm. In the assembly process, the sunshade needs to provide sufficient space for the robotic arm. After the assembly, the sunshade is expanded. Then, the active optical adjustment and optical imaging work are carried out.

The sunshade is required throughout the whole process as follows:

(1) The sunshade module needs to meet the volume, weight, and other limitation requirements of the package when the sunshade is in the cargo ship.
(2) The sunshade module needs to meet the volume and other limitation requirements of the CSS.
(3) The sunshade module needs to meet the volume, weight, and other limitation requirements when the sunshade moves from the inside of the CSS to the outside of the CSS.
(4) The sunshade module needs to provide sufficient volume for the robotic arm during the assembly process.
(5) The sunshade module needs to meet the function of keeping out the stray light.
(6) The sunshade module’s dynamic responses should not affect the imaging of the optical system.

To meet the sixth requirement, vibration isolation devices are installed between the sunshade and the main optical machine. Besides the sixth requirement, other requirements can be reduced to the constraint requirements for the sunshade module in the folded state and deployable state, which can be addressed by a good structural and mechanism design.

### 3.2. Structure Design

The deployable mechanism of the sunshade is made up of a radial deployable mechanism and an axial deployable mechanism. The radial deployable mechanism achieves folding and deployable functions through in-plane rotation. The axial deployable mechanism is a scissor-like element (SLE), which achieves folding and deployment in a straight axial direction.

Because the CSS is in LEO, to effectively block stray light mainly from the sun, the sunshade needs to have a certain angle. Hence, the number of SLEs in the axial deployable mechanism may not be the same.

A single deployable component consists of two sets of driving devices, corresponding to the radial and axial deployable mechanisms. Most of the structures are made of aluminum alloy. Hence, the deployable sunshade assembly structures meet all the requirements proposed in Section 3.1. The next general structure design will adopt a more lightweight structure design.

**Figure 6:**
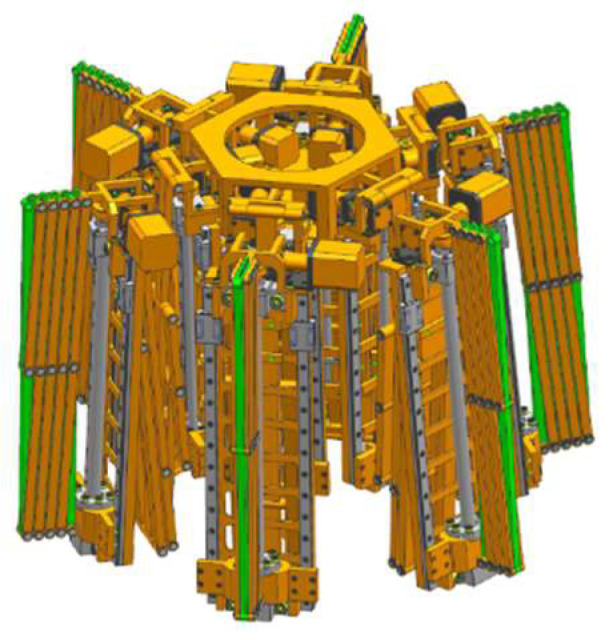
The deployable sunshade assembly structures in the folding state.

**Figure 7:**
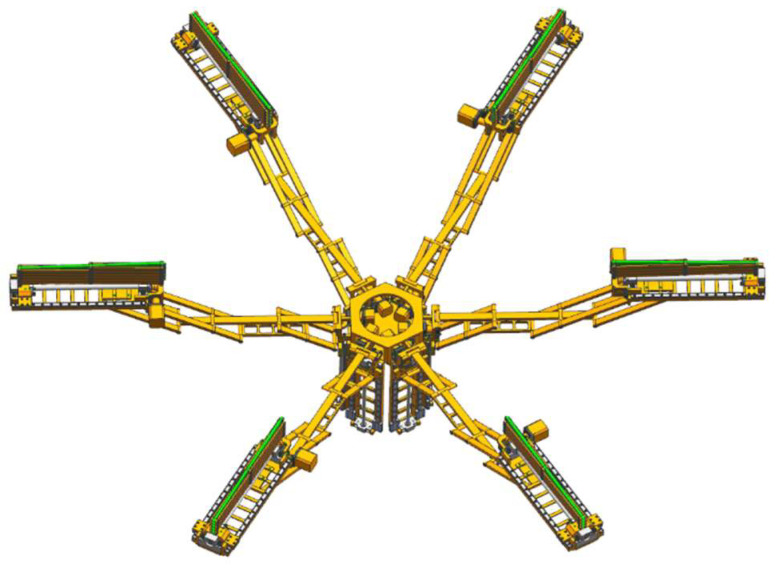
The deployable sunshade assembly structures in a deployable state (The radial deployable mechanism is deployed).

**Figure 8:**
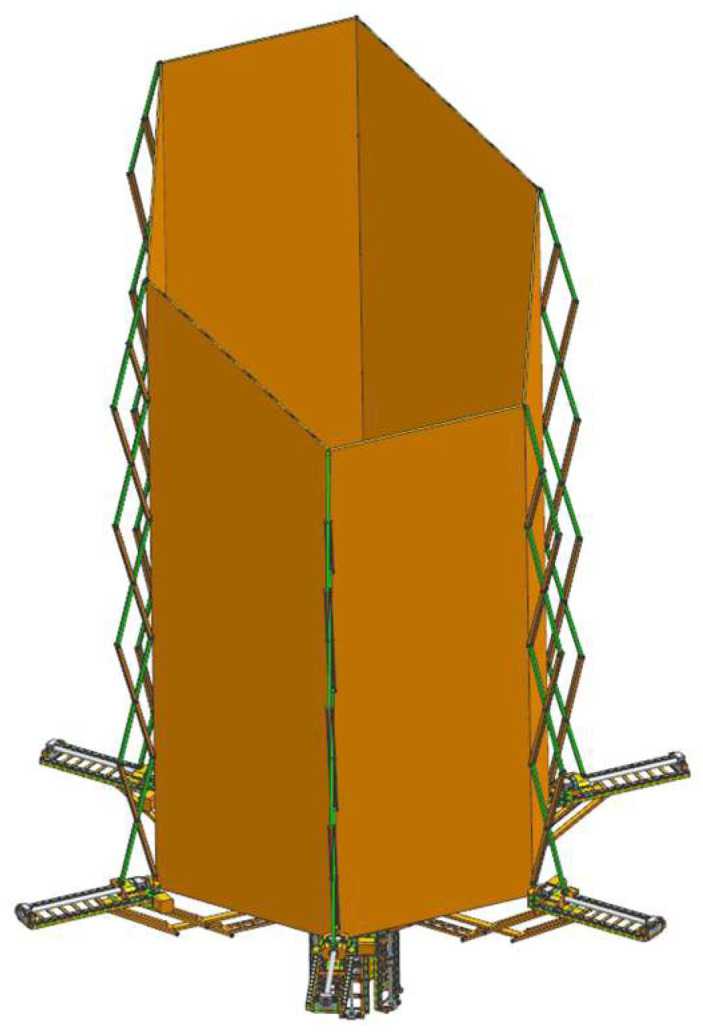
The deployable sunshade assembly structures in a deployable state (The radial and axial deployable mechanisms are deployed).

The deployable sunshade assembly structures are shown in Figure 6, Figure 7 and Figure 8. Figure 6 shows the deployable sunshade assembly structures in the folding state. Figure 7 shows the radial deployable mechanism deployed, and Figure 8 shows the radial and axial deployable mechanisms deployed. To meet the volume and weight requirements of the sunshade, the parameters of the structure need to be adjusted carefully.

### 3.3. Kinematics Analysis

#### 3.3.1. Radial Deployable Mechanism

**Figure 9:**
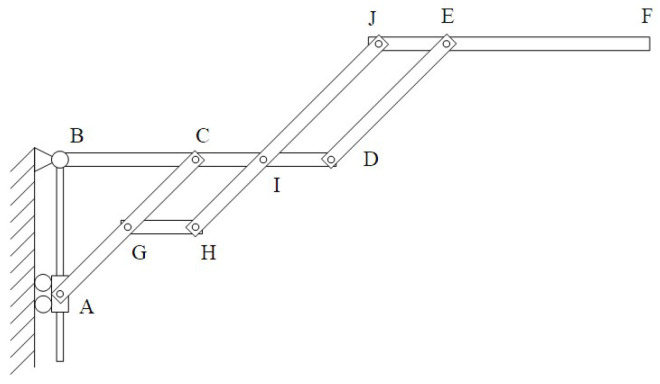
The model of the radial deployable mechanism.

The radial deployable mechanism consists of several radial deployable components. A single radial deployable component is shown in Figure 9. There are ten key points of the single radial deployable component, such as points *A*, *B*, *C*, *D*, *E*, *F*, *G*, *H*, *I*, and *J*. There are six rods, such as rods *AC*, *BD*, *DE*, *GH*, *HJ*, and *JF*. Point *C* is on the rod *BD*. Point *E* is on the rod *JF*. Point *G* is on the rod *AC*. And the point *I* is on the rod *BD*. Rods *AC*, *HJ*, and *DE* are in parallel. Rods *BD*, *GH*, and *JF* are in parallel.

The length of *AB* is *l_c_*. The length of *AC* is *l_b_*. The length of *BC* is *l_a_*. The length of *BD* is *l_d_*. The length of the *CD* is *l_d_* − *l_a_*. The length of *DE* is *l_e_*. The length of *EF* is *l_f_*. The length of *GC* is *l_h_*. The length of *GH* is *l_g_*. The length of the *ID* is *l_d_* − *l_a_*. The length of *JE* is *l_d_* − *l_a_* − *l_g_*. Hence, we can get the angle between rod *BC* and rod *BA* and the angle between rod *AB* and rod *AC*, which can be expressed as

(1) $$\cos∠CBA=\frac{l_{a}^{2}+l_{c}^{2}-l_{b}^{2}}{2l_{a}l_{c}}$$

(2) $$\cos∠BAC=\frac{l_{b}^{2}+l_{c}^{2}-l_{a}^{2}}{2l_{b}l_{c}}$$

Hence, the ten points’ coordinates can be expressed as

(3) $$A\left(0,-l_{c}\right)$$

(4) $$B\left(0,0\right)$$

(5) $$C\left(l_{a}\sin∠CBA,-l_{a}\cos∠CBA\right)$$

(6) $$D\left(l_{d}\sin∠CBA,-l_{d}\cos∠CBA\right)$$

(7) $$E\left(l_{d}\sin∠CBA+l_{e}\sin∠BAC,-l_{d}\cos∠CBA+l_{e}\cos∠BAC\right)$$

(8) $$F\left(l_{d}\sin∠CBA+l_{e}\sin∠BAC+l_{f}\sin∠CBA,-l_{d}\cos∠CBA+l_{e}\cos∠BAC-l_{f}\cos∠CBA\right)$$

(9) $$G\left(l_{a}\sin∠CBA-l_{h}\sin∠BAC,-l_{a}\cos∠CBA-l_{h}\cos∠BAC\right)$$

(10) $$H\left(l_{a}\sin∠CBA-l_{h}\sin∠BAC+l_{g}\sin∠CBA,-l_{a}\cos∠CBA-l_{h}\cos∠BAC-l_{g}\cos∠CBA\right)$$

(11) $$I\left(l_{a}\sin∠CBA+l_{g}\sin∠CBA,-l_{a}\cos∠CBA-l_{g}\cos∠CBA\right)$$

(12) $$J\left(l_{a}\sin∠CBA+l_{g}\sin∠CBA+l_{e}\sin∠BAC,-l_{a}\cos∠CBA-l_{g}\cos∠CBA+l_{e}\cos∠BAC\right)$$

#### 3.3.2. Axial Deployable Mechanism

**Figure 10:**
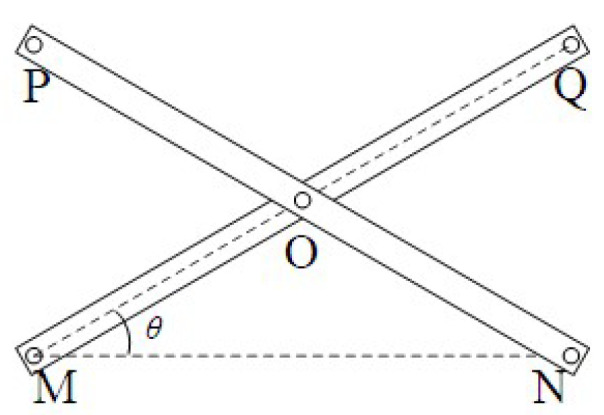
The model of the SLE.

A single axial deployable component consists of several SLEs. Each SLE is the mechanism are shown in Figure 10. There are five key points of the single SLE, such as points *M*, *N*, *O*, *P*, and *Q*. Point *O* is the midpoint of the rod *MQ* and rod *NP*. The length of both *MQ* and *NP* is *l*. The length of both *MO* and *NO* is *l*/2. The distance of *MN* is *x*. *θ* is the angle from *MN* to *MQ*. The angle is positive for anticlockwise. Hence, we can get the angle of the *NMQ*, which can be shown as

(13) $$\cos\theta =\frac{x}{l}$$

The point coordinates of the *i*-th SLE can be expressed as

(14) $$M_{i}\left(0,\left(i-1\right)l\sin\theta \right)$$

(15) $$N_{i}\left(x,\left(i-1\right)l\sin\theta \right)$$

(16) $$O_{i}\left(\frac{1}{2}x,\left(i-\frac{1}{2}\right)l\sin\theta \right)$$

(17) $$P_{i}\left(0,il\sin\theta \right)$$

(18) $$Q_{i}\left(x,il\sin\theta \right)$$

The point velocities of the *i*-th SLE can be expressed as

(19) $${\dot{M}}_{i}=\left(0,\left(i-1\right)\frac{-x}{\sqrt{l^{2}-x^{2}}}\dot{x}\right)$$

(20) $${\dot{N}}_{i}=\left(\dot{x},\left(i-1\right)\frac{-x}{\sqrt{l^{2}-x^{2}}}\dot{x}\right)$$

(21) $${\dot{O}}_{i}=\left(\frac{1}{2}\dot{x},\left(i-\frac{1}{2}\right)\frac{-x}{\sqrt{l^{2}-x^{2}}}\dot{x}\right)$$

(22) $${\dot{P}}_{i}=\left(0,i\frac{-x}{\sqrt{l^{2}-x^{2}}}\dot{x}\right)$$

(23) $${\dot{Q}}_{i}=\left(\dot{x},i\frac{-x}{\sqrt{l^{2}-x^{2}}}\dot{x}\right)$$

The point accelerations of the *i*-th SLE can be expressed as

(24) $${\ddot{M}}_{i}=\left(0,\left(i-1\right)\left(\frac{-l^{2}}{{\left(l^{2}-x^{2}\right)}^{\frac{3}{2}}}{\dot{x}}^{2}+\frac{-x}{\sqrt{l^{2}-x^{2}}}\ddot{x}\right)\right)$$

(25) $${\ddot{N}}_{i}=\left(\ddot{x},\left(i-1\right)\left(\frac{-l^{2}}{{\left(l^{2}-x^{2}\right)}^{\frac{3}{2}}}{\dot{x}}^{2}+\frac{-x}{\sqrt{l^{2}-x^{2}}}\ddot{x}\right)\right)$$

(26) $${\ddot{O}}_{i}=\left(\frac{1}{2}\ddot{x},\left(i-\frac{1}{2}\right)\left(\frac{-l^{2}}{{\left(l^{2}-x^{2}\right)}^{\frac{3}{2}}}{\dot{x}}^{2}+\frac{-x}{\sqrt{l^{2}-x^{2}}}\ddot{x}\right)\right)$$

(27) $${\ddot{P}}_{i}=\left(0,i\left(\frac{-l^{2}}{{\left(l^{2}-x^{2}\right)}^{\frac{3}{2}}}{\dot{x}}^{2}+\frac{-x}{\sqrt{l^{2}-x^{2}}}\ddot{x}\right)\right)$$

(28) $${\ddot{Q}}_{i}=\left(\ddot{x},i\left(\frac{-l^{2}}{{\left(l^{2}-x^{2}\right)}^{\frac{3}{2}}}{\dot{x}}^{2}+\frac{-x}{\sqrt{l^{2}-x^{2}}}\ddot{x}\right)\right)$$

The rotation velocities of the *i*-th SLE can be expressed as

(29) $$\omega_{MQ}=-\omega_{NP}=\frac{-1}{\sqrt{l^{2}-x^{2}}}\dot{x}$$

The rotation accelerations of the *i*-th SLE can be expressed as

(30) $$\alpha_{MQ}=-\alpha_{NP}=\frac{-\ddot{x}\left(l^{2}-x^{2}\right)-x{\dot{x}}^{2}}{{\left(l^{2}-x^{2}\right)}^{\frac{3}{2}}}$$

#### 3.3.3. Structure Parameters

The above equations can be combined to establish the structural kinematics model; the structural boundary constraint equations, such as the constraint boundary equations in the process of axial deployable mechanism, can be established

(31) $$\left\{\begin{array}{l} X_{M}=X_{P}\geq R_{0}\\ Y_{P}=Y_{Q}\geq h_{0} \end{array}\right.$$

where *R*_0_ indicates the radius of the sunshade inscribed circle, and *h*_0_ indicates the height of the sunshade. The constraint boundary equations before the radial deployable process

(32) $$\left\{\begin{array}{l} Y_{Q5}\leq l_{0}\\ Y_{Q6}\leq w_{0}\times \cos30^{\circ } \end{array}\right.$$

where *l*_0_ indicates the length of the storage box, and *w*_0_ indicates the width of the storage box.

The initial structural parameters are obtained through parameter adjustment. Then, the parameters are adjusted based on the 3D model and corrected if there are structural interferences. Finally, the structural design is obtained as shown in Figure 6, Figure 7 and Figure 8.

### 3.4. Force Analyses

The sunshade needs to deploy on the CSS. To verify the feasibility of the motion mechanism, it is necessary to conduct force analyses on the deployment mechanism of the sunshade and determine the motor torque. This section mainly analyzes its development along the optical axis direction as an example. The force analysis by formulations provides a preliminary scheme design and uses multi-dynamic software (UG 10) for simulation to get more accurate data.

#### 3.4.1. The Force Analysis of the Top SLE

**Figure 11:**
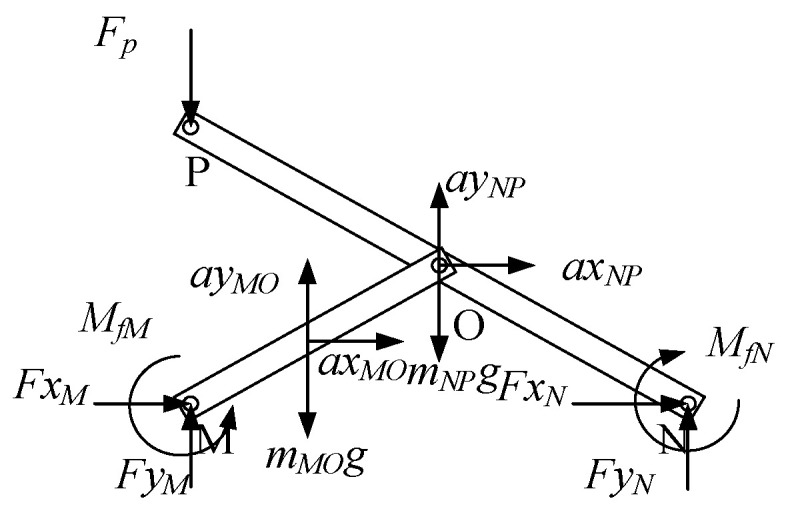
The force analysis of the top SLE.

**Figure 12:**
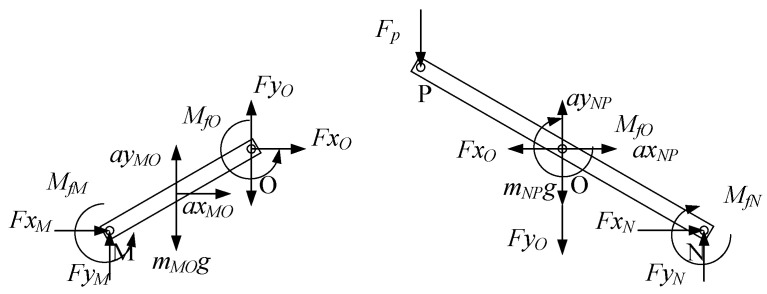
The force analyses of the rod *MO* and rod *NP*.

The force analysis of the top SLE is shown in Figure 11 where a possible external force *F_p_* is applied at point *P*. The force analyses of the rod *MO* and rod *NP* are shown in Figure 12. The calculation formulation can be obtained as follows

(33) $$Fx_{M}+Fx_{O}=m_{MO}ax_{MO}$$

(34) $$Fy_{M}+Fy_{O}=m_{MO}g+m_{AC}ay_{MO}$$

(35) $$\left(Fx_{M}-Fx_{O}\right)\frac{1}{4}l\sin\theta +\left(Fy_{O}-Fy_{M}\right)\frac{1}{4}l\cos\theta +M_{fM}+M_{fO}=Jr_{MO}\omega_{MO}$$

(36) $$Fx_{N}-Fx_{O}=m_{NP}ax_{NP}$$

(37) $$Fy_{N}-Fy_{O}-F_{p}-m_{NP}g=m_{NP}ay_{NP}$$

(38) $$Fx_{N}\frac{1}{2}l\sin\theta +Fy_{N}\frac{1}{2}l\cos\theta +F_{p}\frac{1}{2}l\cos\theta -M_{fO}-M_{fN}=Jr_{NP}\omega_{NP}$$

where, *M_fM_*, *M_fO_*, and *M_fN_* are friction torques caused by friction. They can be calculated by functions as follows

(39) $$\begin{array}{c} M_{fM}=-\mathrm{sgn}\left(\omega_{MQ}\right)\mu \sqrt{F{x_{M}}^{2}+F{y_{M}}^{2}}R\\ M_{fO}=-\mathrm{sgn}\left(\omega_{MQ}\right)\mu \sqrt{F{x_{O}}^{2}+F{y_{O}}^{2}}R\\ M_{fN}=-\mathrm{sgn}\left(\omega_{MQ}\right)\mu \sqrt{F{x_{N}}^{2}+F{y_{N}}^{2}}R \end{array}$$

where *μ* is the coefficient of friction, and *R* is the radius of the pin. Hence, we can get the forces of the points *M*, *N*, and *O*, which are *Fx_M_*, *Fy_M_*, *Fx_N_*, *Fy_N_*, *Fx_O_*, and *Fy_O_*.

#### 3.4.2. The Force Analysis of the Normal SLE

**Figure 13:**
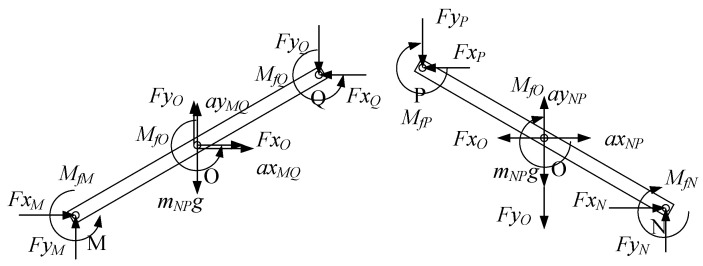
The force analyses of the rod *MQ* and the rod *NP*.

The force analyses of the rod *MQ* and the rod NP are shown in Figure 13. The calculation formulation can be obtained as follows

(40) $$Fx_{M}+Fx_{O}-Fx_{Q}=m_{MQ}ax_{MQ}$$

(41) $$Fy_{M}+Fy_{O}-Fy_{Q}-m_{MQ}g=m_{MQ}ay_{MQ}$$

(42) $$Fx_{M}\frac{1}{2}l\sin\theta -Fy_{M}\frac{1}{2}l\cos\theta +Fx_{Q}\frac{1}{2}l\sin\theta -Fy_{Q}\frac{1}{2}l\cos\theta +M_{fM}+M_{fO}+M_{fQ}=Jr_{MQ}\omega_{MQ}$$

(43) $$Fx_{N}-Fx_{O}-Fx_{P}=m_{NP}ax_{NP}$$

(44) $$Fy_{N}-Fy_{O}-Fy_{P}-m_{NP}g=m_{NP}ay_{NP}$$

(45) $$Fx_{N}\frac{1}{2}l\sin\theta +Fy_{N}\frac{1}{2}l\cos\theta +Fx_{P}\frac{1}{2}l\sin\theta +Fy_{P}\frac{1}{2}l\cos\theta -M_{fN}-M_{fO}-M_{fP}=Jr_{NP}\omega_{NP}$$

where *M_fQ_* and *M_fP_* are friction torques caused by friction. They can be calculated by functions as follows

(46) $$\begin{array}{c} M_{fP}=-\mathrm{sgn}\left(\omega_{MQ}\right)\mu \sqrt{F{x_{P}}^{2}+F{y_{P}}^{2}}R\\ M_{fQ}=-\mathrm{sgn}\left(\omega_{MQ}\right)\mu \sqrt{F{x_{Q}}^{2}+F{y_{Q}}^{2}}R \end{array}$$

*Fx_P_*, *Fy_P_*, *Fx_Q_*, and *Fy_Q_* are known quantities. They are reaction forces. Hence, we can get the forces of the points *M*, *N*, and *O*, which are *Fx_M_*, *Fy_M_*, *Fx_N_*, *Fy_N_*, *Fx_O_*, and *Fy_O_*.

#### 3.4.3. Force Analysis Results

**Figure 14:**
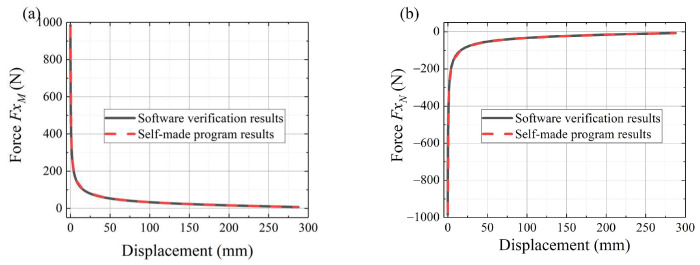
The force analysis results: (**a**) horizontal force of point M, (**b**) horizontal force of point N.

To calculate the forces of point *M*, point *N*, and point *O*, we program according to the above equations. To verify the accuracy of the calculation results, we use multi-body dynamics software for verification. Both models are in the gravity. Figure 14a,b shows the horizontal forces of point *M* and point *N* without friction, respectively. The black solid line represents the software verification results. The red dashed line represents the calculation results of the self-made program. It shows that the red line results are consistent with the black line results, which proves the validity of the force analysis. From simulation data and the structure design, the motor torque can be determined.

**Figure 15:**
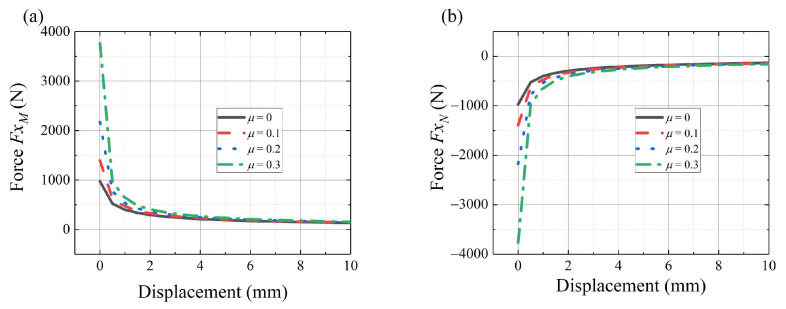
The force analysis results with friction: (**a**) horizontal force of point M, (**b**) horizontal force of point N.

In practice, the effect of friction can greatly influence the deployable mechanism. The functions above can be used to complete the preliminary design. Figure 15a,b shows the horizontal forces of point *M* and point *N* with friction, respectively. It shows different forces with different friction parameters. At the starting position of the movement, the driving force peaks and rapidly decreases. Figure 15 only shows the beginning of the movement.

**Figure 16:**
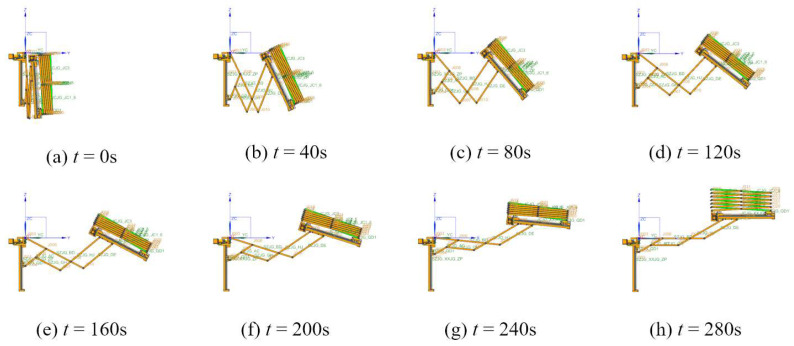
Motion simulation of the radial deployable mechanism.

**Figure 17:**
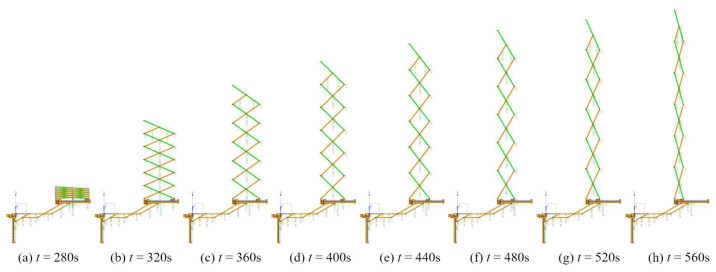
Motion simulation of the axial deployable mechanism.

**Figure 18:**
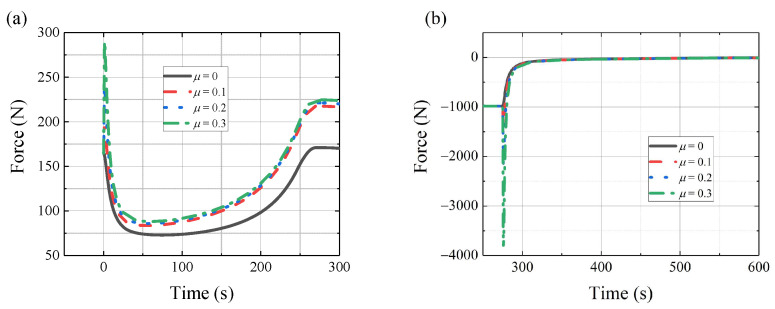
Forces of the radial and axial deployable mechanism: (**a**) forces of radial deployable mechanism, (**b**) forces of axial deployable mechanism.

Multi-body dynamic software is used to finish the dynamic simulation. The motion processes are shown in Figure 16 and Figure 17. The radial deployable motion and the axial deployable motion are completed in turn. Figure 18a,b shows the driving forces of the radial deployable mechanism and the axial deployable mechanism with friction, respectively.

Table 1 shows the peak value of the forces. It shows that the maximum relative error is approximately 3.2% with *μ* = 0.3. From the multi-body dynamic software solutions, when considering friction with *μ* = 0.3, the force of the radial deployable increases about 173% from 168 N to 290 N at the beginning and about 132% from 171 N to 225 N, and the force of the axial deployable increase around 395% from 984 N to 3887 N. The friction coefficient is assumed to be 0.3. From simulation data and the structure design, the motor torque can be determined.

### 3.5. Vibrational Modes

**Figure 19:**
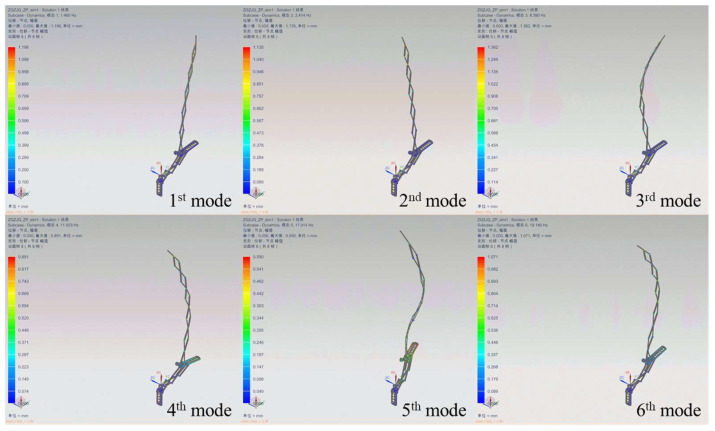
The vibrational modes of the single deployable component.

The vibrational modes of the single deployable component are shown in Figure 19. It shows the first to the sixth mode shapes. The numerical results of the vibrational modes are shown in Table 2. The minimum frequency of the structure is 1.46 Hz. For the 1st mode and 2nd mode, the mode shapes mainly swing in the two horizontal directions, respectively.

**Figure 20:**
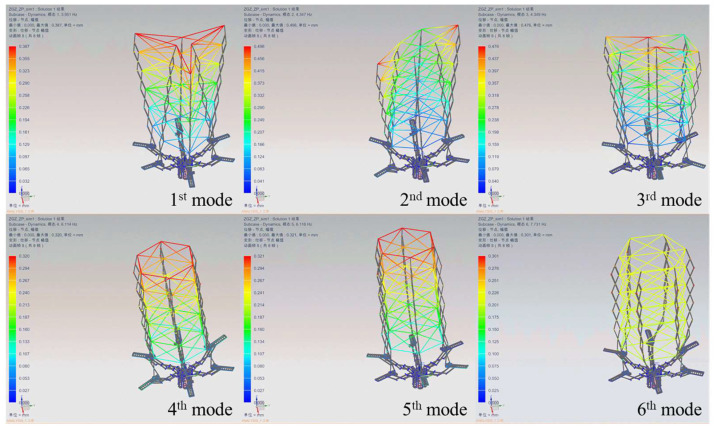
The vibrational modes of sunshade deployable components.

The sunshade usually consists of several deployable components. The vibrational modes of the sunshade are shown in Figure 20. It shows the first to the sixth mode shapes. The numerical results of the vibrational modes are shown in Table 2. The minimum frequency of the structure is 3.951 Hz. By increasing the constraints, the 1st mode and 2nd mode frequencies increase. Figure 20 shows more mode shapes—such as the sixth mode is the twisting mode.

The current simulation results show that there should be a vibration isolation device between the telescope optical system and the sunshade. The performance of the vibration isolation device determines the optical system metrics. Also, the natural frequency of the sunshade deployable components can still be improved by structure design, such as the constraints between single components.

## 4. Preliminary Ground Experiment

**Figure 21:**
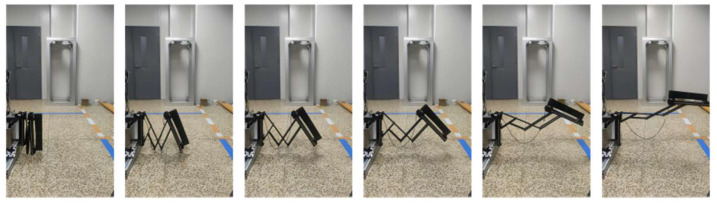
The experimental process of the radial deployable mechanism.

**Figure 22:**
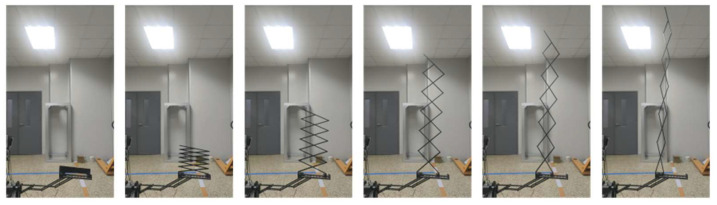
The experimental process of the axial deployable mechanism.

A single deployable component preliminary ground experiment is shown in Figure 21 and Figure 22. Figure 21 and Figure 22 show the experimental process of the radial deployable mechanism and the axial deployable mechanism, respectively. The deployable component does not unload gravity. After the radial deployable expansion, the axial deployable mechanism system’s benchmark plane has an angle of inclination. Hence, the axial deployable process is in a tilted line.

The stepper motors of the single deployable component adopt constant speed control. The subsequent actual motor control process will depend on the velocity requirement of the sunshade and the folding form of the sunshade. The preliminary ground experiment shows the feasibility of the design.

## 5. Conclusions

In this paper, the deployable sunshade of an OAST is studied. The requirements of the sunshade are proposed. A sunshade deployable mechanism component that meets the requirements was designed. To determine the structural parameters, the kinematics models are studied, such as the radial deployable mechanism kinematics model and the axial deployable mechanism kinematics model. To ensure the motor torque, the force analyses of the SLEs are conducted. Numerical results show that the initial force reached 3887 N with *μ* = 0.3 for the axial deployable mechanism and 290 N with *μ* = 0.3 for the radial deployable mechanism. The vibrational modes of the single deployable component and the sunshade are analyzed. Numerical results show that the 1st mode of the single deployable component is 1.46 Hz and the 1st mode of the sunshade is 3.951 Hz. In addition, a preliminary ground experiment was conducted, which demonstrated the feasibility of the design.

## Figures and Tables

**Table 1:** The max forces of point M and point N.

*μ*	Max Forces (N)			
	Software Verification Results		Self-Developed Program Results	
	M Point	N Point	M Point	N Point
0	984.19556	−984.19556	972.49583	−972.49583
0.1	1380.55898	−1380.55898	1393.08162	−1393.08162
0.2	2200.74375	−2200.74375	2173.03178	−2173.03178
0.3	3887.88354	−3887.88354	3764.02054	−3764.02054

**Table 2:** The frequencies of deployable component’s vibrational modes.

Modes	Frequency (Hz)	
	Single Deployable Component	Sunshade Deployable Components
1st mode	1.460	3.951
2nd mode	2.414	4.347
3rd mode	8.380	4.349
4th mode	11.923	6.114
5th mode	17.014	6.116
6th mode	19.189	7.731
7th mode	22.904	17.065
8th mode	35.232	17.284
9th mode	41.369	17.289
10th mode	43.037	17.730

## Data Availability

Data are contained within the article.
